# Exploring the Effects on Lipid Bilayer Induced by Noble Gases *via* Molecular Dynamics Simulations

**DOI:** 10.1038/srep17235

**Published:** 2015-11-25

**Authors:** Junlang Chen, Liang Chen, Yu Wang, Xiaogang Wang, Songwei Zeng

**Affiliations:** 1 School of Sciences, Zhejiang A & F University, Lin’an 311300, China; 2 School of Information and Industry, Zhejiang A & F University, Lin’an 311300, China; 3 Shanghai Applied Radiation Institute, Shanghai University, Shanghai 200444, China; 4 Zhejiang Provincial Key Laboratory of Chemical Utilization of Forestry Biomass, Zhejiang A & F University, Lin’an 311300, China

## Abstract

Noble gases seem to have no significant effect on the anesthetic targets due to their simple, spherical shape. However, xenon has strong narcotic efficacy and can be used clinically, while other noble gases cannot. The mechanism remains unclear. Here, we performed molecular dynamics simulations on phospholipid bilayers with four kinds of noble gases to elucidate the difference of their effects on the membrane. Our results showed that the sequence of effects on membrane exerted by noble gases from weak to strong was Ne, Ar, Kr and Xe, the same order as their relative narcotic potencies as well as their lipid/water partition percentages. Compared with the other three kinds of noble gases, more xenon molecules were distributed between the lipid tails and headgroups, resulting in membrane’s lateral expansion and lipid tail disorder. It may contribute to xenon’s strong anesthetic potency. The results are well consistent with the membrane mediated mechanism of general anesthesia.

The noble gases (He, Ne, Ar, Kr and Xe), except helium and probably neon, can cause general anesthesia^1^. However, their narcotic potencies vary widely in degree. For example, xenon is an excellent anesthetic and has already been clinically used in surgical operations^2^,^3^,^4^, though the specific molecular mechanisms of general anesthesia are still under debate.

The mechanisms of general anesthesia are divided into membrane and protein hypotheses^5^,^6^,^7^,^8^. According to the membrane mechanism, the entering of anesthetic in membrane leads to the change of the physical properties of membrane (such as lipid order and lateral pressure), which further affects the function of ion channels embedded within the membrane, and may finally cause general anesthesia^9^,^10^,^11^. This hypothesis is supported by Meyer and Overton based on the correlation between anesthetic potency and lipid solubility, which is known as the Meyer-Overton correlation: the anesthetic potency is proportional to the lipid/water partition coefficient^12^,^13^. Although this rule is true for a wide range of anesthetics including noble gases, it suffers from several weaknesses. For example, stereoisomers of an anesthetic have very different narcotic potency, whereas their oil/gas partition coefficients are similar^14^.

Therefore, researchers have resorted to protein hypothesis and focused on the binding of anesthetics to trans-membrane ion channels^15^,^16^,^17^,^18^. Recently, experiments have shown that N-methyl-D-aspartic acid (NMDA) receptor and γ-aminobutyric acid (GABA_A_) receptor are molecular targets for general anesthesia^19^,^20^,^21^,^22^,^23^. Also, xenon can inhibit the NMDA receptor while mutations of the NMDA receptor reduce the anesthetic sensitivity^24^. Other anesthetics, however, interact with neurotransmitter receptors very differently compared with xenon, such as isoflurane, which anesthetic target is GABA_A_ receptor^25^. There is still no consensus in the protein mechanism of general anesthesia.

Since the electrons in outer shells of noble gases are saturated and spherically symmetric, noble gases have apparently minimal capacities to bind with a putative action site. More recently, researchers resume their interests in the membrane mediated mechanism of general anesthesia^26^,^27^. Yamamoto *et al*. performed molecular dynamics simulation to study the diffusive nature of xenon within the lipid bilayer^28^. They found that xenon molecules are concentrated at the center of the bilayer and lipid tail order increases under extremely high pressure (100 ~ 500bar). They proposed that pressure reveral of general anesthesia originated from the recovery of membrane disorder previously caused by xenon. Booker *et al*. simulated six xenon concentrations (0 ~ 3 xenon molecules per lipid) and reported significant changes in lipid bilayer properties induced by xenon, including bilayer thickness increasing and modulation of lateral pressure profile^29^. They therefore indicated that these changes may directly affect the gated ion channel function that finally lead to general anesthesia. Through measuring the electric polarizabilities of noble gases in the pure lipid membrane, Sierra-Valdez and Ruiz-Suarez found that the increasing membrane disorder induced by noble gases is He, Ar, Kr and Xe in turn^30^. They suggested that general anesthesia depends on the ability of certain molecules to increase the general disorder of the membrane. Weinrich and co-worker’s experiment demonstrated that inhalation anesthetics could affect the lipid environment of membrane proteins, which supported the membrane-mediated mechanism^31^.

In this paper, we investigate the interactions of four kinds of noble gases (Ne, Ar, Kr and Xe) with 1-palmitoyl-2-oleoyl phosphatidylethanolamine (POPE) bilayer using molecular dynamics simulations. Noble gases show the wide effects on bilayer structure, including increasing area per lipid and lipid tail diorder. The sequence of changes on membrane from weak to strong is Ne, Ar, Kr and Xe, the same order as their relative narcotic potencies. The results sufficiently support the membrane mediated mechanism of general anesthesia.

## Methods

Molecular dynamics (MD) simulations were performed on POPE bilayers in aqueous solution with or without noble gas molecules. The lipid bilayer was composed of 256 POPE molecules (128 molecules in each leaflet) and was fully hydrated with 40 water molecules per lipid. Four kinds of noble gases (Ne, Ar, Kr and Xe) and five concentrations (0, 1, 1.5, 2 and 3 noble gas molecules per lipid) were simulated. The initial configurations were generated from the pure hydrated system by replacing water molecules with noble gas molecules randomly according to the fixed concentration. We then minimized their energy using the conjugate gradient method and continued to perform 200 ns MD simulations for each system.

All MD simulations were performed in the isothermal-isobaric (NPT) ensemble using the Gromacs package 4.5.5^32^. The force field parameters for POPE lipids were taken from Berger *et al*.^33^,^34^. Noble gas atoms were treated as uncharged Lennard-Jones (LJ) spheres and their ver der Waals (vdW) parameters were taken from UFF force field^35^. The temperature was kept stable at 310 K using the V-rescale thermostat and the pressure was controlled semi-isotropically at 1 bar by a Berendsen barostat^36^,^37^. The particle-mesh Ewald method (PME) was used to calculate the long-range electrostatic interactions, whereas the vdW interactions were treated with smooth cutoff at a distance of 10 Å^38^,^39^. Water was represented by the simple point-charge (SPC) model^40^. Bond lengths within the DPPC lipids and water molecules were constrained by the LINCS and the SETTLE algorithms, which allowed an integration time of 2 fs. We applied periodic boundary conditions in all three directions and used the last 20 ns trajectory of each system to analyze the properties of noble gases and POPE bilayers.

To demonstrate that the systems had been well equilibrated at *t* = 180 ns, we compared the noble gas density profiles of the first, middle and last 10 ns trajectories, as shown in Figure S1, and monitored the time evolution of area per lipid and lipid volume in Figure S2, which confirmed that the systems had already reached equilibrium from about *t* = 100 ns.

## Results and Discussion

### Noble gas molecules partitioning

At dynamical equilibrium, noble gas molecules (Ne, Ar, Kr and Xe) within the bilayer account for 77.3%, 85.2%, 91.0% and 95.7% of the total, initially localized in the aqueous phase. According to the Meyer-Overton correlation, the greater the lipid/water partitioning coefficient is, the stronger its anesthetic potency is. Therefore, our simulation results are in good agreement with this rule. Although the partitioning percentage presents a little fluctuation because of different gas concentration, the results still obey the correlation after equilibrium.

**Figure 1:**
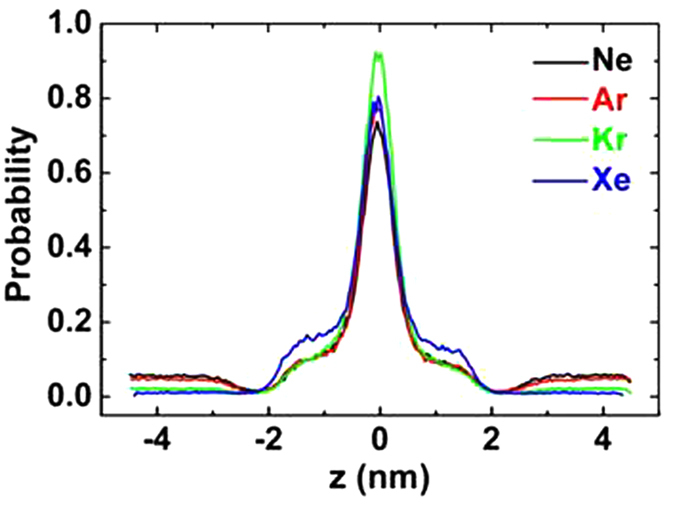
Density of the *z* direction of four kinds of noble gases (Ne, black, Ar, red, Kr, green and Xe blue), where the z axis is normal to the lipid bilayer. The center of the lipid bilayer is fixed at *z* = 0 nm.

**Figure 2:**
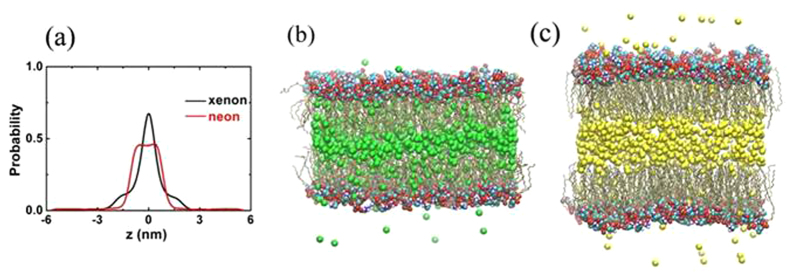
Results of 3 noble gas molecules per lipid system. (**a**) Density distribution. (**b**,**c**) are final snapshots at *t* = 200 ns. Carbon (cyan), oxygen (red), nitrogen (blue) and phosphorous (tan) in head groups are shown using spheres while the lipid tails are shown as dynamic-bonds. Xenon and neon molecules are depicted as green and yellow balls. Water molecules are omitted for clarity. The images are created by VMD software.

The partitioning of the noble gases within the bilayer can be more quantitatively illustrated by the noble gas density distributions. Figure 1 shows the densities of the z direction of four kinds of noble gases. The peak of each curve is at the center of the bilayer for all systems, indicating that noble gas molecules are preferentially aggregated at the center of the membrane, since there is an interspace between the upper and lower leaflets. This localization of gas molecules is almost the same as previous simulations performed by Stimson *et al*. and Yamamoto *et al*.^28^,^29^,^41^. However, the probability of xenon localizing near the lipid head groups is about 10% higher than those of the other three gases (Ne, Ar and Kr), which is of great importance to explain xenon’s strong narcotic efficacy. Yamamoto proposed that the narcotic function of xenon will be decreased when xenon molecules move to the hydrophobic core of the lipid bilayer and are jammed there under high pressures. Other anesthetics, such as halothane, diethyl ether, enflurane and ethanol, prefer two different positions, namely the center of the membrane and the hydrocarbon region, close to the polar headgroups^5^,^42^,^43^. Therefore, we suggest that the narcotic potency of xenon is much stronger because more xenon molecules than other noble gases are distributed between the lipid tails and headgroups. This speculation can be further confirmed at higher noble gas concentration (3 noble gas atoms per lipid). As shown in Fig. 2, about 90% neon atoms are congested in the middle of the membrane and stretch the bilayer at the normal direction, while about 40% xenon atoms diffuse near the hydrocarbon region and expand the membrane at the lateral direction.

### Effects of noble gases on membrane structure

**Figure 3:**
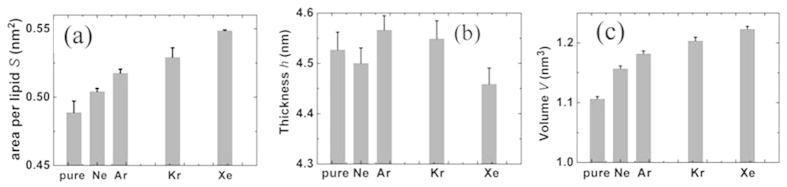
Effects of noble gas molecules on bilayer structural properties. (**a**) Area per lipid, (**b**) thickness and (**c**) volume per lipid. Error bars mean the root mean square deviations.

Figure 3 shows area per lipid *S*, thickness *h* and volume *V* in the five independent systems. The area per lipid is defined as the area of the *xy*-plane of the simulation box divided by the number of lipids per leaflet. The thickness of lipid bilayer *h* is defined as the distance between the average *z*-position of the phosphorus atom in the two layers. And the volume per lipid is defined as *V* = *Sh*/2. In general, the three parameters containing noble gases are bigger than those of pure hydrated membrane. Furthermore, it is interesting to find that the values of *S* and *V* rise gradually in the order of Ne, Ar, Kr and Xe, which is in agreement with the sequence of their narcotic potencies from weak to strong. The area per lipid and volume with xenon increase at the most by 12.2% and 9.54%, respectively. However, the thickness *h* does not hold this trend, especially xenon, as shown in Fig. 3b. For example, the thickness with argon increases 0.87%, while *h* with xenon decreases 0.33%, compared with pure membrane. The thickness *h* is mainly determined by two factors: the distributions of gas molecules and lipid tail ordering. As mentioned above, xenon within the membrane leads to its lateral expansion more than normal stretch.

**Figure 4:**
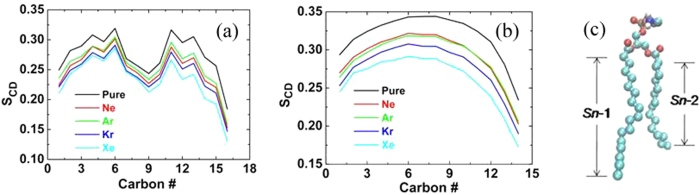
Lipid tail order parameters of (**a**) *sn*-1 chain and (**b**) *sn*-2 chain. (**c**) Atomic structure of POPE (cyan, red, blue and tan balls represent carbon, oxygen, nitrogen and phosphorus atoms, respectively).

The localization of noble gases in the hydrophobic core of the bilayer has substantial effects on the ordering of the lipid acyl chains. Figure 4 shows the two lipid tail (*sn*-1 and *sn*-2) deuterium order parameters of five systems. The deuterium order is defined by


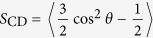


where *θ* is the relative angle between the CD bond vector and the bilayer normal and the brackets denote averaging over molecules and simulation time^44^. The order parameter provides a quantitative measure of the alignment of the lipid tails. The value of *S*_CD_ with noble gases is lower than that of purely hydrated membrane system because gas molecules diffuse in the lipid bilayer and collide with the lipid tails. Interestingly, it is found that the sequence of effects on lipid tail ordering induced by noble gases is Ne, Ar, Kr and Xe, which is also in agreement with their narcotic potency. This sequence is also in accordance with the previous experiment^30^. The value of *S*_CD_ with xenon decreases the most significantly, about 16.2%.

## Conclusions

By extensive MD simulations of POPE membranes in aqueous solution with four kinds of noble gases, we find that the sequence of effects on POPE from weak to strong is Ne, Ar, Kr and Xe, which is in agreement with their relative narcotic potencies. The localization of noble gases within the membrane leads to a relative increase in area, volume per lipid and decrease in lipid tail ordering, which can increase membrane fluidity and depress the membrane gel-liquid crystalline phase transition temperature^29^.

Due to its deep potential well and wide collision radius, xenon is prone to diffuse between the lipid tails and headgroups than other noble gases, inducing more lateral expansion of the membrane and lipid tail disorder. Of the four noble gases, the distribution of xenon within the membrane is the most similar to that of other anesthetics, such as chloroform and enflurane. Xenon’s vigorous effects on membrane structure and localization therein contribute to its strong anesthetic efficacy. These results are in favor of the membrane mediated mechanism of general anesthesia.

## Additional Information

**How to cite this article**: Chen, J. *et al*. Exploring the Effects on Lipid Bilayer Induced by Noble Gases via Molecular Dynamics Simulations. *Sci. Rep*. **5**, 17235; doi: 10.1038/srep17235 (2015).

## Supplementary Material

Supplementary Information
