# Supplementary material for: Exploring the Effects on Lipid Bilayer Induced by Noble Gases via Molecular Dynamics Simulations
Source: Sci Rep. 2015 Nov 25;5:17235. doi: 10.1038/srep17235 (PMC4658558; doi:10.1038/srep17235)
Supplement: Supplementary Information [file srep17235-s1.doc]

Supplementary material to

"Exploring the Effects on Lipid Bilayer Induced by Noble Gases *via* Molecular Dynamics Simulations"

by

Junlang Chen, Liang Chen, Yu Wang, Xiaogang Wang, Songwei Zeng


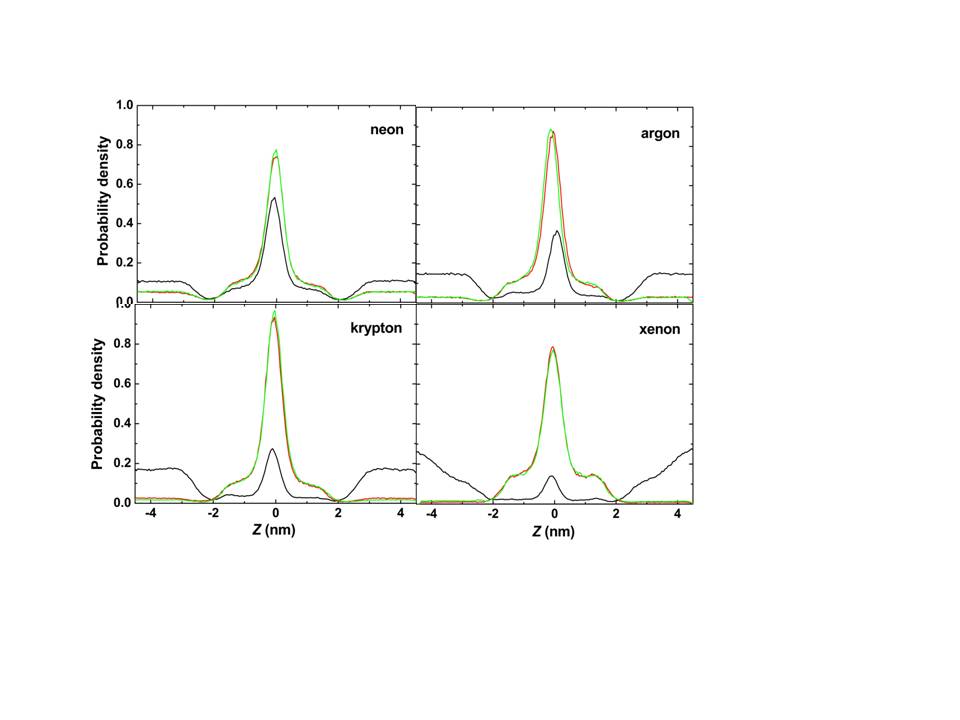


Figure S1. Probability density profiles of noble gas molecules along the membrane normal axis *z*. The center of the bilayer is set as z=0 nm. Black, red and green lines are the densities in the first, middle and last 10 ns trajectories, respectively.


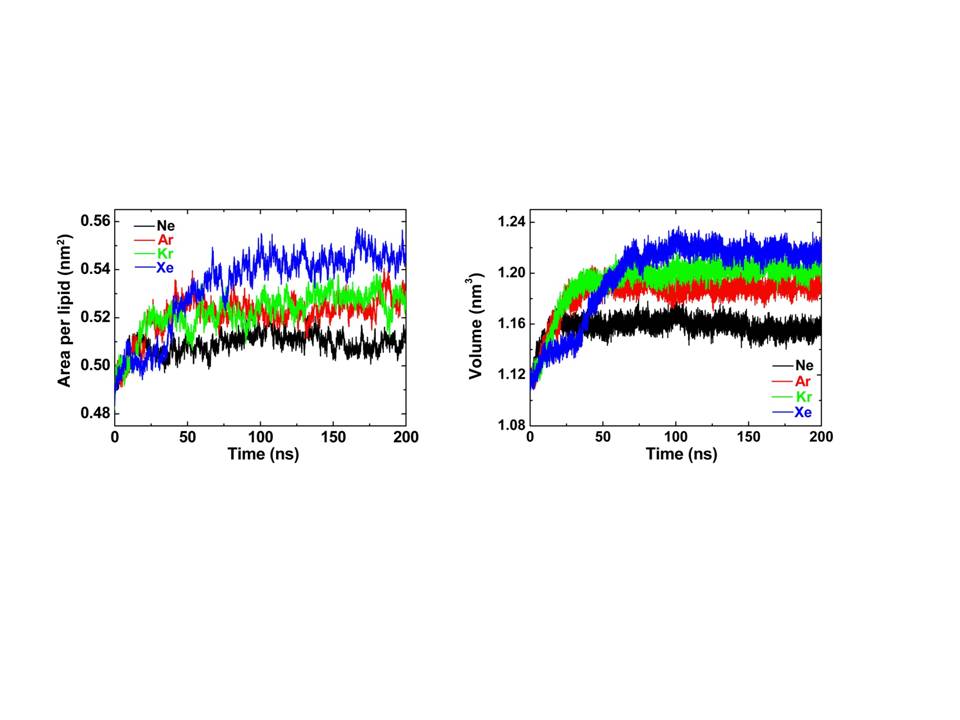


Figure S2. Time evolutions of area per lipid (left) and volume per lipid (right).

Figure S1 shows that the densities in the middle and last 10 ns trajectories are well consistent with each other, indicating that the systems have reached equilibriums from about *t*=100 ns, which is further confirmed by the time evolutions of area per lipid or volume per lipid in Figure S2.
